# Development of Novel Prime-Boost Strategies Based on a Tri-Gene Fusion Recombinant *L. tarentolae* Vaccine against Experimental Murine Visceral Leishmaniasis

**DOI:** 10.1371/journal.pntd.0002174

**Published:** 2013-04-18

**Authors:** Noushin Saljoughian, Tahereh Taheri, Farnaz Zahedifard, Yasaman Taslimi, Fatemeh Doustdari, Azam Bolhassani, Delaram Doroud, Hiva Azizi, Kazem Heidari, Mohammad Vasei, Nabiollah Namvar Asl, Barbara Papadopoulou, Sima Rafati

**Affiliations:** 1 Molecular Immunology and Vaccine Research Laboratory, Pasteur Institute of Iran, Tehran, Iran; 2 Department of Quality Control, Research and Production Complex, Pasteur Institute of Iran, Tehran, Iran; 3 Research Centre in Infectious Disease, CHUL Research Centre and Department of Microbiology, Infectious Disease and Immunology, Laval University, Quebec, Canada; 4 Department of Epidemiology and Biostatistics, School of Public Health, Tehran University of Medical Sciences, Tehran, Iran; 5 Department of Pathology, Shariati Hospital, Tehran University of Medical Sciences, Tehran, Iran; 6 Department of Laboratory of Animal Sciences, Pasteur Institute of Iran, Tehran, Iran; Universidad Autónoma de Yucatán, Mexico

## Abstract

Visceral leishmaniasis (VL) is a vector-borne disease affecting humans and domestic animals that constitutes a serious public health problem in many countries. Although many antigens have been examined so far as protein- or DNA-based vaccines, none of them conferred complete long-term protection. The use of the lizard non-pathogenic to humans *Leishmania (L.) tarentolae* species as a live vaccine vector to deliver specific *Leishmania* antigens is a recent approach that needs to be explored further. In this study, we evaluated the effectiveness of live vaccination in protecting BALB/c mice against *L. infantum* infection using prime-boost regimens, namely Live/Live and DNA/Live. As a live vaccine, we used recombinant *L. tarentolae* expressing the *L. donovani* A2 antigen along with cysteine proteinases (CPA and CPB without its unusual C-terminal extension (CPB^-CTE^)) as a tri-fusion gene. For DNA priming, the tri-fusion gene was encoded in pcDNA formulated with cationic solid lipid nanoparticles (cSLN) acting as an adjuvant. At different time points post-challenge, parasite burden and histopathological changes as well as humoral and cellular immune responses were assessed. Our results showed that immunization with both prime-boost A2-CPA-CPB^-CTE^-recombinant *L. tarentolae* protects BALB/c mice against *L. infantum* challenge. This protective immunity is associated with a Th1-type immune response due to high levels of IFN-γ production prior and after challenge and with lower levels of IL-10 production after challenge, leading to a significantly higher IFN-γ/IL-10 ratio compared to the control groups. Moreover, this immunization elicited high IgG1 and IgG2a humoral immune responses. Protection in mice was also correlated with a high nitric oxide production and low parasite burden. Altogether, these results indicate the promise of the A2-CPA-CPB^-CTE^-recombinant *L. tarentolae* as a safe live vaccine candidate against VL.

## Author Summary

Visceral leishmaniasis (VL) is the most severe form of leishmaniasis and has emerged as an opportunistic infection in HIV-1 infected patients in many parts of the world. Drug-resistant forms have developed so emergence and increased the need for advanced preventive strategies. Using live avirulent organisms as a vaccine has been proven to be more effective than other regimens. The lizard protozoan parasite *Leishmania tarentolae* is considered as nonpathogenic to humans. In our previous work, a recombinant *L. tarentolae* strain expressing the amastigote-specific *L. donovani* A2 antigen as a vaccine candidate elicited protection against *L. infantum* challenge in mice. Furthermore, combinations of CPA/CPB cysteine proteinases were more protective against visceral and cutaneous *Leishmania* infections than the individual forms. Herein, we used DNA/Live and Live/Live prime-boost vaccination strategies against visceral leishmaniasis in BALB/c mice consisting of the A2-CPA-CPB^-CTE^ tri-fusion genes formulated with cationic solid lipid nanoparticles and a recombinant *L. tarentolae* expressing the tri-fusion. Assessments of cytokine production, humoral responses, parasite burden and histopathological studies support that the recombinant *L. tarentolae* A2-CPA-CPB^-CTE^ candidate vaccine elicits a protective response against visceral leishmaniasis in mice and represents an important step forward in the development of new vaccine combinations against *Leishmania* infections.

## Introduction

Leishmaniasis is a vector-borne disease caused by different *Leishmania* species that ranges from self-limiting cutaneous leishmaniasis to fatal visceral leishmaniasis (VL) and is endemic in 88 tropical and subtropical countries [1]. VL is caused by members of the *L. donovani* complex with a wide and growing prevalence and incidence (http://www.who.int/en/) [2] and is considered as the most severe form of leishmaniasis and is often fatal, if left untreated [3], [4]. *L. infantum* is responsible for VL in the Mediterranean basin, extending to several Middle East and Asian countries [5]. VL has emerged as an opportunistic infection in HIV-1 infected patients in many parts of the world [6], [7], [8]. Currently, prophylactic or therapeutic vaccines are not available and the control of the disease depends exclusively on chemotherapy. However, drug-resistant forms have developed from current chemotherapeutic interventions [9], [10]. Therefore, much attention has been given to improve vaccination strategies. Although induction of lifelong protection against reinfection in recovered individuals demonstrates that a protective vaccine can be achieved, an effective vaccine against human leishmaniasis has not been yet developed [11]. Various vaccination strategies have been explored against experimental leishmaniasis, with particular emphasis on their efficacy against CL rather than VL [12], [13]. First-generation anti-leishmanial vaccines based on live parasites (leishmanization) are the only successful intervention against leishmaniasis [14], [15]. However, leishmanization was largely abandoned due to safety issues. The development of second-generation vaccines for *Leishmania* included recombinant proteins, polyproteins, DNA vaccines, liposomal formulation, and dendritic cell vaccine delivery systems [16], [17], [18]. Also, multicomponent vaccines have been shown to protect against VL in experimental infection systems [17], [19], [20], [21], [22], [23]. Furthermore, it has been reported that persistence of a small number of live parasites is essential for maintaining durable immunity [24], [25]. The only way to meet this requirement is by using attenuated live vaccines. Attenuated strains based either on long-term *in vitro* culturing [26] or culturing under drug pressure [27] or on selection for temperature sensitivity [28] and chemical mutagenesis [29] are not easily applicable to human use because there is always a risk of reversion of the organism to its virulent state. Alternatively, approaches based on the genetic attenuation of *Leishmania* genes encoding virulence factors or enzymes responsible for their synthesis and genes essential for intracellular survival have been reported [30], [31], [32], [33], [34], [35], [36]. Other approaches to develop live attenuated parasites as VL vaccines have utilized nonpathogenic *Leishmania* species, an approach comparable to the use of BCG as a vaccine against *Mycobacterium tuberculosis* infections.

Among different species of *Leishmania*, the lizard protozoan parasite *L. tarentolae* has never been found associated with any form of human leishmaniasis and is therefore considered nonpathogenic [37]. Whilst *L. tarentolae* is capable of infecting mammalian cells and transforming into amastigote-like forms, it is not however able to persist long enough within macrophages [38], [39]. The use of *L. tarentolae* as a vaccine vector to deliver specific *Leishmania* antigens mimicking live infection has also been explored. In a previous study, the *L. donovani* amastigote-specific A2 antigen was expressed in *L. tarentolae*, which lacks this protein [40], [41] and used as a vaccine strain in an experimental mouse model. Vaccination protected susceptible mice against *L. infantum* challenge and was associated with the production of high levels of IFN-γ production [42].

It has been reported that sera from either cured or active cases of cutaneous and visceral leishmaniasis patients recognize the recombinant cysteine proteinases CPA (rCPA) and CPB (rCPB) of *L. major* and *L. infantum* [43], [44] that are members of the papain superfamily [45]. CPA (type II) and CPB (type I) are expressed at higher levels in amastigotes [45] and stationary-phase promastigotes [46]. An unusual C-terminal extension (CTE) of 110 amino acids distinguishes CPB enzyme from the other CPs in the papain superfamily [47], [48]. Immunization with CTE also displayed both type 1 and 2 immune signatures in experimental murine model of *L. infantum* infection and therefore is not protective as a vaccine candidate [49]. Furthermore, we have demonstrated that the combination of CPA/CPB and CPA/CPB^-CTE^ is more protective against visceral and cutaneous leishmanial infections than the individual forms [50], [51], [52], [53], [54]. Despite the proven antigenicity and immunogenicity of these DNA vaccine candidates, their largest drawback is the inefficient intracellular delivery of pDNA causing low levels of gene expression, which in turn limits the resulting immune responses [55]. In our previous studies, cationic solid-lipid nanoparticles (cSLN) as an effective delivery system has exhibited considerable low cytotoxicity, and it was able to protect pDNA in a DNase I challenge assay [54], [56].

Here, we use the A2-CPA-CPB^-CTE^ tri-gene fusion as a DNA vaccine formulated with cSLN and also a recombinant *L. tarentolae* expressing the tri-gene fusion as a live vaccination strategy against visceral leishmaniasis in two-modalities, namely DNA/Live and Live/Live vaccination in BALB/c mice. We demonstrate that prime-boost strategies harboring recombinant *L. tarentolae*-based vaccines represent a promising immunization approach against *Leishmania* infections.

## Materials and Methods

### Reagents

All solutions were prepared using MilliQ ultrapure (Milli-QSystem, Millipore, Molsheim, France) and apyrogenic water to avoid surface-active impurities. Cetyl palmitate, Tween 80 and cholesterol were purchased from Merck (Darmstadt, Germany). G418, N-[1-(2,3-Dioleoyloxy) propyl]-N,N,Ntrimethylammonium chloride (DOTAP), Sodium dodecyl sulfate (SDS) were purchased from Sigma-Aldrich (Sigma, Deisenhofen, Germany). The materials applied for PCR, enzymatic digestion and agarose gel electrophoresis were acquired from Roche Applied Sciences (Mannheim, Germany). Cell culture reagents including M199 medium, HEPES, L-glutamine, adenosine, hemin, gentamicin, RPMI and Schneider were sourced from Sigma (Darmstadt, Germany) and Gibco (Gibco, Life Technologies GmbH, Karlsruhe, Germany), respectively. Fetal Calf Sera (FCS) was purchased from (Gibco, Life Technologies GmbH, Karlsruhe, Germany).

### DNA Constructs

The *A2* gene (with Kozak sequence) was digested from pUC57 vector (synthesized by Shine Gene Molecular Biotech, Inc) with *Eco*RI and *Hind*III restriction sites. After sequence confirmation, the A2 fragment was subcloned into the *Eco*RI and *Hind*III sites of vector pGEM7zf(+) (Promega). The CPA fragment was amplified from pGEM-CPA using Taq DNA Polymerase (Roche, Germany) and the following primers: (forward, 5′-GTTAAGCTTCGCCCCCAGTGGTGT-3′) including *Hind*III restriction site (underlined); and (reverse, 5′-TTTGCTAGCCTAGGCCGTTGTCGT-3′) including *Nhe*I restriction site (underlined). Then, the PCR-amplified *CPA* gene was cloned into *Hind*III and *Nhe*I sites of pAT153 vector (Boca Scientific). The CPB^-CTE^ fragment was amplified from pGEM-CPB using Taq DNA Polymerase (Roche, Germany) and the following primers: (forward, 5′-AATGCTAGCGATGCGGTGGACTGG-3′) harboring *Nhe*I restriction site (underlined); and (reverse, 5′-ACTGGATCCCACATGCGCGGA-3′) including *Bam*HI restriction site (underlined). The PCR-amplified CPB^-CTE^ gene was cloned into the *Nhe*I and *Bam*HI sites of pAT153 vector downstream the *CPA* gene. Then CPA-CPB^-CTE^ fusion gene was digested with *Hind*III and *Bam*HI restriction sites and cloned downstream of the *A2* gene in the pGEM7zf(+) vector. After sequence confirmation, the A2-CPA-CPB^-CTE^ fusion gene was subcloned into the *Eco*RI and *Bam*HI sites of vector pEGFP-N3 upstream of the *GFP* gene to generate pEGFP-A2-CPA-CPB^-CTE^, and the correct insert orientation was confirmed by restriction analysis. Then, the A2-CPA-CPB^-CTE^-GFP fragment was subcloned into the *Bgl*II and *Not*I sites of vector pLEXSY-NEO2 (EGE-233, Jena Bioscience, Germany) to generate pLEXSY-A2-CPA-CPB^-CTE^-GFP. Also the A2-CPA-CPB^-CTE^ fragment was subcloned into the *Eco*RI and *Bam*HI sites of vector pcDNA3.1(−) (Invitrogen, Germany) to generate pcDNA-A2-CPA-CPB^-CTE^ as a DNA vaccine. pcDNA-A2-CPA-CPB^-CTE^ plasmid was purified by ion exchange chromatography with Endofree Mega kit (QIAGEN, Germany).

### Parasite Growth and Transfections

The *L. tarentolae* Tar II (ATCC 30267) strain was grown at pH 7.2 and 26°C in M199 medium (Sigma) supplemented with 5% heat-inactivated fetal calf serum (FCS, Gibco), 40 mM HEPES, 0.1 mM adenosine, 5 µg/ml hemin and 50 µg/ml gentamicin. For transfection, 4×10^7^ log-phase parasites were washed and re-suspended in 300 µl of electroporation buffer (21 mM HEPES, 137 mM NaCl, 5 mM KCl, 0.7 mM Na2HPO4, 6 mM glucose; pH 7.5) and mixed with 50 µl H_2_O containing 15 µg of linearized pLEXSY-A2-CPA-CPB^-CTE^-GFP with *Swa*I, stored on ice for 10 min, and electroporated (Bio-Rad Gene Pulser Ecell, Germany) at 450 V and 500 µF as described previously [57]. In brief, the electroporated promastigotes were then incubated for 24 h in M199 10% medium at 26°C without any drug (Neomycin or G418, Gibco, Germany), and plated on solid media (2% of Noble agar and 2XM199 10% (vol/vol), Sigma, Germany) containing 50 µg/ml of G418. The growth of cells highly resistant to Neomycin was observed after 7–10 days. Clones were selected on Noble agar plates and further propagated in liquid M199 10% medium in the absence of G418. Expression of EGFP in *Leishmania* promastigotes was evaluated by Epi-fluorescent microscopy for up to 3 months (Nikon, E 200, ACT-1 software, Digital sight Camera, Japan).

### Confirmation of the A2-CPA-CPB^-CTE^-GFP Fusion Gene Expression in Transgenic *L. tarentolae*

Integration of the expression cassette into the *ssu* locus was confirmed by diagnostic PCR using genomic DNA of transgenic strains as a template extracted by GF-1 Genomic DNA extraction kit (Vivantis, Malaysia). We performed diagnostic PCR (annealing temperature 60°C) with *ssu* forward primer F3001 (5′-GATCTGGTTGATTCTGCCAGTAG-3′) and reverse primer A1715 hybridizing within the 5′UTR of the target gene (5′-TATTCGTTGTCAGATGGCGCAC-3′) according to the LEXSY Kit protocol (Jena bioscience, Germany). Primer pairs including one primer hybridizing within the expression cassette and one primer hybridizing to the *ssu* sequence not present in the plasmid were used. Integration of the expression cassette into the *ssu* locus yielded a 1 kb fragment that was not obtained in the control reactions with the genomic DNA of *L. tarentolae* wild type.

### Southern Blot Analysis

For Southern blot analysis, 5 µg of transgenic *L. tarentolae*-GFP and wild type *L. tarentolae* genomic DNA were digested with the appropriate restriction enzymes (BglII/NotI). DNA was then resolved on 0.7% agarose before being separated and transferred onto membrane according to standard procedures [58]. The membrane was then UV-crosslinked prehybridized with Church mix buffer (7% SDS, 0.5 M NaPi, 1 mM EDTA, 1% BSA) for 1 to 2 h. For probe synthesis, 100 ng of the *GFP* ORF was used by incorporating radiolabeled dCTP using Klenow enzyme. The reaction was finished by addition of 1 µl EDTA (0.5 M) and subsequently, the probe was purified by passing through a Sephadex resin. The membrane was then washed once with Wash buffer 1 (2× SSC, 0.5% SDS) at 25°C for 30 min, then 2 times at 65°C with Wash buffer 2 (1× SSC, 0.1% SDS) for 15 min. The membrane was then exposed for overnight and developed by a Konicka Minolta developer.

### RNA Extraction and Reverse-Transcription PCR

RNA samples were extracted from promastigote forms using RNeasy kit (Qiagen) and treated with RNase-free DNase for 30 min at 37°C to eliminate any remaining DNA. cDNA synthesis was performed using the Qiagen Omniscript RT Kit from 1 µg of RNA. To detect the A2, CPA, CPB^-CTE^ and GFP cDNAs, PCR reactions were carried out using specific primer pairs to amplify each gene separately.

### Fluorescence Microscopy and Flow Cytometry Analysis

Promastigote forms of *L. tarentolae*-A2-CPA-CPB^-CTE^-GFP were examined for GFP expression by Epi-fluorescence microscopy. Promastigotes were centrifuged in 3000 rpm for 15 min and after washing once with PBS, cells were re-suspended in PBS and mounted on microscope slides. Expression of EGFP protein was evaluated by Epi-fluorescent microscopy (Nikon, E 200, ACT-1 software, Digital sight Camera, Japan). Also wild type (as negative control) and GFP expressing promastigote forms were analyzed for EGFP expression using flow cytometry. Parasites at two different growth phases (logarithmic and stationary phases) were centrifuged at 3000 rpm for 15 min, washed and then re-suspended at 10^6^ cell/ml in PBS and stored on ice. Cells were analyzed on a FACS caliber flow cytometer (BD: Becton Dickinson, Franklin Lakes, NJ) equipped with a 15 mV, 488 nm, air-cooled argon ion laser. 50,000 events were recorded and EGFP expression in transgenic *L. tarentolae* was measured in comparison with wild type (WT) parasites and *L. tarentolae*-GFP expressing parasites.

### Western Blot Analysis

Promastigote forms of *L. tarentolae*-A2-CPA-CPB^-CTE^-GFP were harvested by centrifugation at 3000 rpm for 15 min and washed in PBS. The pellets were immediately lysed in 2× SDS-PAGE sample buffer (4.5 mM Tris-HCl, pH 6.8, 10%, v/v glycerol, 2%, w/v SDS, 5%, v/v 2-mercaptoethanol, 0.05%, w/v bromophenol blue) on ice and then boiled for 5 min. Samples were then loaded on a 15% SDS-PAGE. The gels were transferred onto a nitrocellulose membrane using a Bio-Rad wet blotting system and incubated with blocking solution (PBS with 0.1% Tween 20 and 2.5% BSA) for 1 h. Washing was performed 3 times with 0.1% Tween 20 in PBS, and blots were incubated overnight with previously prepared rabbit anti-CPB polyclonal antibodies [43], [52] as the first antibody at 1∶50 dilution. The membranes were washed three times and incubated for 90 min with peroxidase-conjugated goat anti mouse IgG (1∶5000, Sigma) assecondary antibodies. Unbound secondary antibodies were removed by washing as described above. Diaminobenzidine tetrahydrochloride (DAB, Sigma) were used as the substrate to detect the desired bands of the protein.

### Mice Infections

Six-week-old female BALB/c mice were obtained from the breeding stock maintained at the Pasteur Institute of Iran. The *L. infantum* strain MCAN/ES/98/LLM-877 was kindly provided by WHO collaborating center for leishmaniasis, Servicio de Parasitología, Centro Nacional de Microbiología, Instituto de Salud Carlos III, Madrid, Spain and kept virulent by continuous passage in hamsters. Amastigotes were isolated from the spleen of infected hamsters and cultured in NNN media in presence of 100 µg/ml of gentamicin. Stationary-phase promastigotes were harvested after 5–6 days by centrifugation (270×g, 5 min, 4°C), washed three times in PBS (8 mM Na_2_HPO_4_, 1.75 mM KH_2_PO_4_, 0.25 mM KCl and 137 mM NaCl) and re-suspended at a concentration of 2×10^8^ parasites/ml. This preparation was frozen and thawed (F/T) 10 times using liquid N2 and a 37°C water-bath and protein concentration was determined by bicinchoninic acid reagent (BCA, PIERCE, Chemical Co. Rochford III). For infection, virulent promastigotes were harvested in the stationary phase, washed in PBS and injected (10^7^) by the lateral tail vein into BALB/c mice.

### Ethics Statement

All mouse experiments including maintenance, animals' handling program and blood sample collection were approved by Institutional Animal Care and Research Advisory Committee of Pasteur Institute of Iran (Education Office dated January, 2008), based on the Specific National Ethical Guidelines for Biomedical Research issued by the Research and Technology Deputy of Ministry of Health and Medicinal Education (MOHME) of Iran (issued in 2005).

### Immunization Schedules and Parasite Challenge

Two independent immunization experiments were carried out in five groups of mice (n = 15 per treatment at each time point) and all tests were done in duplicate or triplicate (number of mice per group/time point n = 2–3). Results are shown as mean±S.E. of measures obtained from 4–6 mice in different groups. Group 1 (DNA cSLN/Live) immunized with pcDNA-A2-CPA-CPB^-CTE^-cSLN (50 µg of pcDNA-A2-CPA-CPB^-CTE^ formulated by cSLN nanoparticles as a chemical delivery as previously described [59] as a prime and with 2×10^7^ recombinant *L. tarentolae* A2-CPA-CPB^-CTE^ as a boost; group 2 (*L. tarentolae* Live A2-CPA-CPB^-CTE^/*L. tarentolae* Live A2-CPA-CPB^-CTE^) vaccinated with 2×10^7^ recombinant *L. tarentolae*-A2-CPA-CPB^-CTE^ as prime and boost; group 3 (PBS as a control); group 4 [(empty vector pcDNA-cSLN (prime)/Live *L. tarentolae* wild type (boost) as a control)]; and group 5 (*L. tarentolae* Live/*L. tarentolae* Live) vaccinated with 2×10^7^ *L. tarentolae* wild type as prime and boost and used as a control. All groups were immunized via footpad. Booster immunization was carried out 3 weeks following the prime immunization. Three weeks after the last immunization, all animals were challenged with 10*^7^* stationary phase *L. infantum* promastigotes by lateral tail vein.

### Determination of Ag-Specific Antibody Responses

Serum samples were analyzed by ELISA for specific antibodies including IgG1 and IgG2a against either rA2, rCPs or *Leishmania* F/T at two different time points: before challenge and 5 weeks after challenge. Briefly, 96-well plates (Greiner) were coated with rA2, rCPA and rCPB or *L. tarentolae*-A2-CPA-CPB^-CTE^ or *L. infantum* F/T, all at10 µg/ml, overnight at 4°C. Plates were blocked with 100 µl of 1% BSA in PBS at 37°C for 2 h to prevent nonspecific binding. Sera were added (with dilution of 1∶100) and incubated 2 h at 37°C. After three washes, Goat Anti-Mouse IgG1-HPR (1∶10,000, Southern Biotech, Canada) or Goat Anti-Mouse IgG2a -HPR (1∶10,000, Southern Biotech, Canada) were added and incubated for 2 h at 37°C. After four washes, plates were incubated for 30 min at 37°C with Peroxidase Substrate System (KPL, ABTS) as substrate. Reactions were stopped with 1% SDS and the absorbance was measured at 405 nm.

### Cytokine Assays

Three mice from each group were sacrificed before challenge and also at 4 and 8 weeks after challenge and spleens were homogenized. After red blood cell lysis using ACK lysis buffer (0.15 M NH_4_Cl, 10 mM KHCO_3_ and 0.1 mM Na_2_EDTA), splenocytes were washed and re-suspended in complete RPMI medium (supplemented with 5% FCS, 1% l-glutamine, 1% HEPES, 0.1% 2ME, 0.1% gentamicin). Cells were then seeded at a density of 3.5×10^6^ cells/ml in the presence of rA2 (10 µg/ml), rCPA (10 µg/ml) and rCPB (10 µg/ml), or *L. infantum* F/T (25 µg/ml), or *L. tarentolae*-A2-CPA-CPB^-CTE^ F/T (25 µg/ml), or medium alone. Concanavalin A (Con A; 5 µg/ml) was also used in all experiments as the positive control. Plates were incubated for 24 h for IL-2 measurement and 5 days for IFN-γ and IL-10 measurement at 37°C in 5% CO2 humidified atmosphere. The IL-2, IFN-γ and IL-10 production in supernatants of splenocyte cultures was measured by sandwich ELISA kits (R&D, Minneapolis, MN, USA), according to the manufacturer's instructions. The minimum detectable dose of mouse IFN-γ, IL-10 and IL-2 is typically less than 2, 4 and 7 pg/mL, respectively. All experiments were run in duplicates.

### Nitric Oxide Assay

Nitrite release was determined in 5-day stimulated splenocytes supernatant at 8 weeks after challenge. In this case, as described in Section 2.13, 100 µl of 5-day incubated culture splenocytes supernatant was collected from each well and subsequently mixed with an equal volume of Griess reagent [0.1 N (1-napthyl) ethylenediamine dihydrochloride, 1% sulfanil amide in 5% H_3_PO_4_] incubated 10 min at RT. Absorbance of the colored complex was determined at 550 nm. The nitric oxide concentration of each corresponding sample was extrapolated from the standard curve plotted with sodium nitrite serial dilution in culture medium.

### Determination of Parasite Burden

Two mice from each group were sacrificed at 2, 4, 8 and 12 weeks after challenge and parasite burden was determined as follows. A piece of spleen and liver was excised, weighed and then homogenized with a tissue grinder in 2 ml of Schneider's *Drosophila* medium (Sigma, Germany) supplemented with 20% heat-inactivated fetal calf serum and gentamicin (0.1%). Under sterile conditions, serial dilutions ranging from 1 to 10^−20^ were prepared in wells of 96 well microtitration plates. After 7 and 14 days of incubation at 26°C, plates were examined with an inverted microscope at a magnification of 40×. The presence or absence of mobile promastigotes was recorded in each well. The final titer was the last dilution for which the well contained at least one motile parasite. The number of parasites per gram was calculated in the following way: parasite burden = −log_10_ (parasite dilution/tissue weight) [60], [61].

Furthermore, real time PCR was used to quantify parasite burden in spleen and liver 4 weeks after challenge for vaccinated groups (G1 and G2) and PBS control group (G3). Two mice from each group were sacrificed and genomic DNA was extracted from 10 mg of spleen and 30 mg of liver tissues using DNeasy Blood & Tissue kit (Qiagen). Two set of primers targeting a region of kinetoplastid minicircle DNA of *L. infantum* named as RV1 and RV2 (forward: 5′-CTTTTCTGGTCCCGCGGGTAGG-3′; reverse: 5′-CCACCTGGCCTATTTTACACCA-3′) were used [62]. Absolute copy number of the target sequence was measured using Applied Biosystem 7500 real time PCR system. *L. infantum* genomic DNA was used in 10-fold dilutions corresponding to 2×10^5^ parasites and used in real time PCR to draw the standard curve. For quantification of parasites in tissues, 300 ng of DNA was subjected to the reaction containing 5 pmol of each forward and reverse primers, 12.5 µl Qiagen QuantiFast SYBR Green Master Mix in total volume of 25 µl. All reactions were performed in duplicate. Conditions for PCR amplification were as follows: 95°C for 10 min; 40 cycles consisting of 95°C for 15 s, 58°C for 30 s, and 72°C for 40 s. Specific amplification of the target region was confirmed by gel electrophoresis of the PCR products.

### Histopathological Studies

Liver, spleen and bone marrow tissues of two animals from each group at 4, 8, and 14 weeks after challenge were fixed by 10% neutral-buffered formalin for 24 h, dehydrated by immersion in increasing concentrations of ethanol (70%, 95%, and then 100%) and then xylene was added before embedding in paraffin wax. Four-micrometer-thick slides were prepared from paraffin blocks and were stained with hematoxylin and eosin (H&E) method. The slides were examined with an Olympus microscope (BX41) and photos were prepared by a DP11 digital camera (Olympus). The slides were reviewed by a pathologist who was not aware of the original treatment of the groups. The parenchyma of the liver was assessed for hepatocyte damage including fatty change, hydropic changes, cholestasis, liver cell necrosis and regenerative changes. The portals were assessed for degree of inflammation and interface hepatitis. Both areas were evaluated for presence of granuloma, neutrophils, plasma cells and lymphocytes as well as intracellular parasites. The micro-architecture of spleen composed of lymphoid follicles, splenic cord, and parafollicular area were evaluated. Presence of granuloma, giant cells and neutrophils were also assessed.

### Statistical Analysis

Statistics were performed using Graph-Pad Prism 5.0 for Windows (Graphpad Software Inc 2007, San Diego, Calif., USA) as well as SPSS version 18. All the data were analyzed with one way ANOVA (Multiple-comparison Tukey post Hoc test) and when required with a Student's t-test. The correlation between the ratio of IFN-γ/IL-10 production and differences in parasite burden at weeks 4 and 8 were calculated using Spearman Correlation method (2 tailed). The area under the curve (AUC) of parasite burden graphs was calculated using Graph-Pad Prism 5.0 software program. The results were considered statistically significant when p<0.05.

## Results

### Generation of a Recombinant *L. tarentolae* Strain Expressing the A2-CPA-CPB^-CTE^-EGFP tri-Fusion Gene

**Figure 1:**
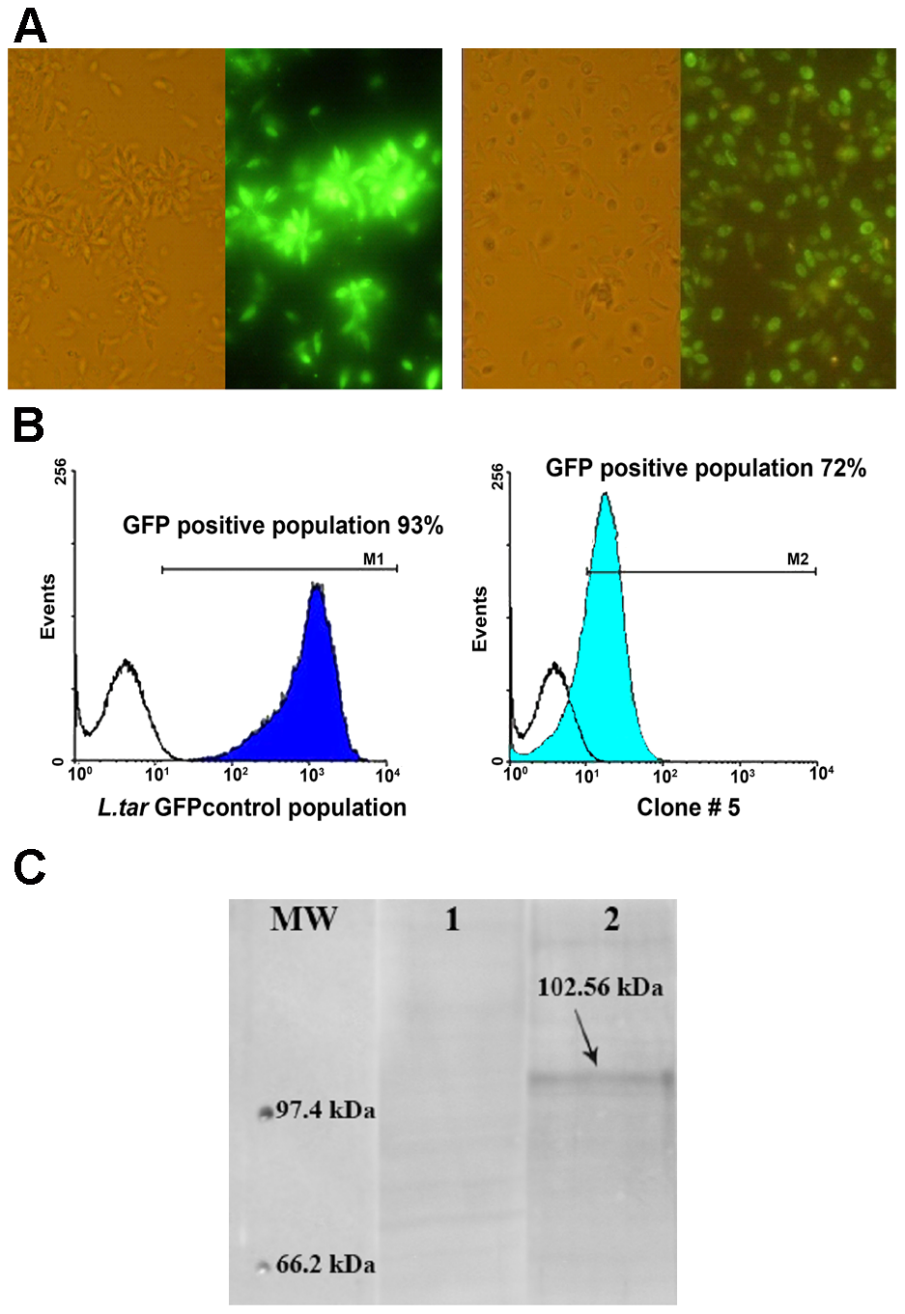
Expression of the A2-CPA-CPB^-CTE^-EGFP tri-fusion gene by *L.tarentolae*. (A) Expression of EGFP by recombinant *L. tarentolae*-EGFP promastigotes (left) and *L. tarentolae*-A2-CPA-CPB^-CTE^-EGFP promastigotes (right) before and after glinting of fluorescence. (B) Percentage of the EGFP positive population in *L. tarentolae* transfected with either pLEXSY-EGFP (left) or pLEXSY-A2-CPA-CPB^-CTE^-EGFP (clone #5, right) as determined by flow cytometry. (C) Western blot analysis for evaluating expression of the A2-CPA-CPB^-CTE^-EGFP fusion protein. A 102.56 kDa band corresponding to the A2-CPA-CPB^-CTE^-EGFP protein was detected in the recombinant *L. tar*-A2-CPA-CPB^-CTE^-EGFP by western blotting using an anti-CPB antibody. No band was seen in lane 1 representing a negative control (*L. tarentolae* wild type).

Recombinant *L. tarentolae* stably expressing the *L. donovani* A2 antigen along with *L. infantum* cysteine proteinases CPA and CPB without its unusual C-terminal extension (CPB^-CTE^) as a tri-fusion gene (A2-CPA-CPB^-CTE^) together with the *EGFP* gene were generated by introducing the linearized pLEXSY-A2-CPA-CPB^-CTE^-EGFP vector into the 18S rRNA *ssu* locus of *L. tarentolae* (Fig. S1A), as indicated in Materials and Methods. Specific targeting of the expression cassette into the *ssu* locus was confirmed both by genomic PCR (Fig. S1B) and by Southern blot analysis using an EGFP-specific probe (Fig. S1C). Amplification of each gene from cDNA resulted in a PCR product of the expected size, hence confirming the expression of A2-CPA-CPB^-CTE^-EGFP by *L. tarentolae* at the mRNA level (Fig. S1D). Expression of EGFP in *L. tarentolae*-A2-CPA-CPB^-CTE^-EGFP and *L. tarentolae*-EGFP parasites was confirmed by fluorescence microscopy (Fig. 1A) and also by fluorescence-activated cell sorting (FACS) analysis (Fig. 1B). The percentage of *L. tarentolae*-A2-CPA-CPB^-CTE^-EGFP-expressing parasites was decreased in logarithmic phase (72%) as compared to *L. tarentolae*-EGFP (93%) with decreasing in mean fluorescent intensities (MFI, Fig. 1B) that may be due to the addition of the tri-fusion genes before EGFP gene which possibly alters translation of EGFP. The A2-CPA-CPB^-CTE^-EGFP expression was assessed by western blot analysis. As shown in Figure 1C, an immunoreactive band of 102.56 kDa was detected in *L. tarentolae* transgenic parasites using an anti-CPB antibody. Altogether, these results are consistent both with a proper integration of the fused A2-CPA-CPB^-CTE^-EGFP gene into *L. tarentolae* and with its constitutive expression.

### Immunization with Live Recombinant *L. tarentolae*-A2-CPA-CPB^-CTE^ Protects Mice against *L. infantum* Infectious Challenge

**Figure 2:**
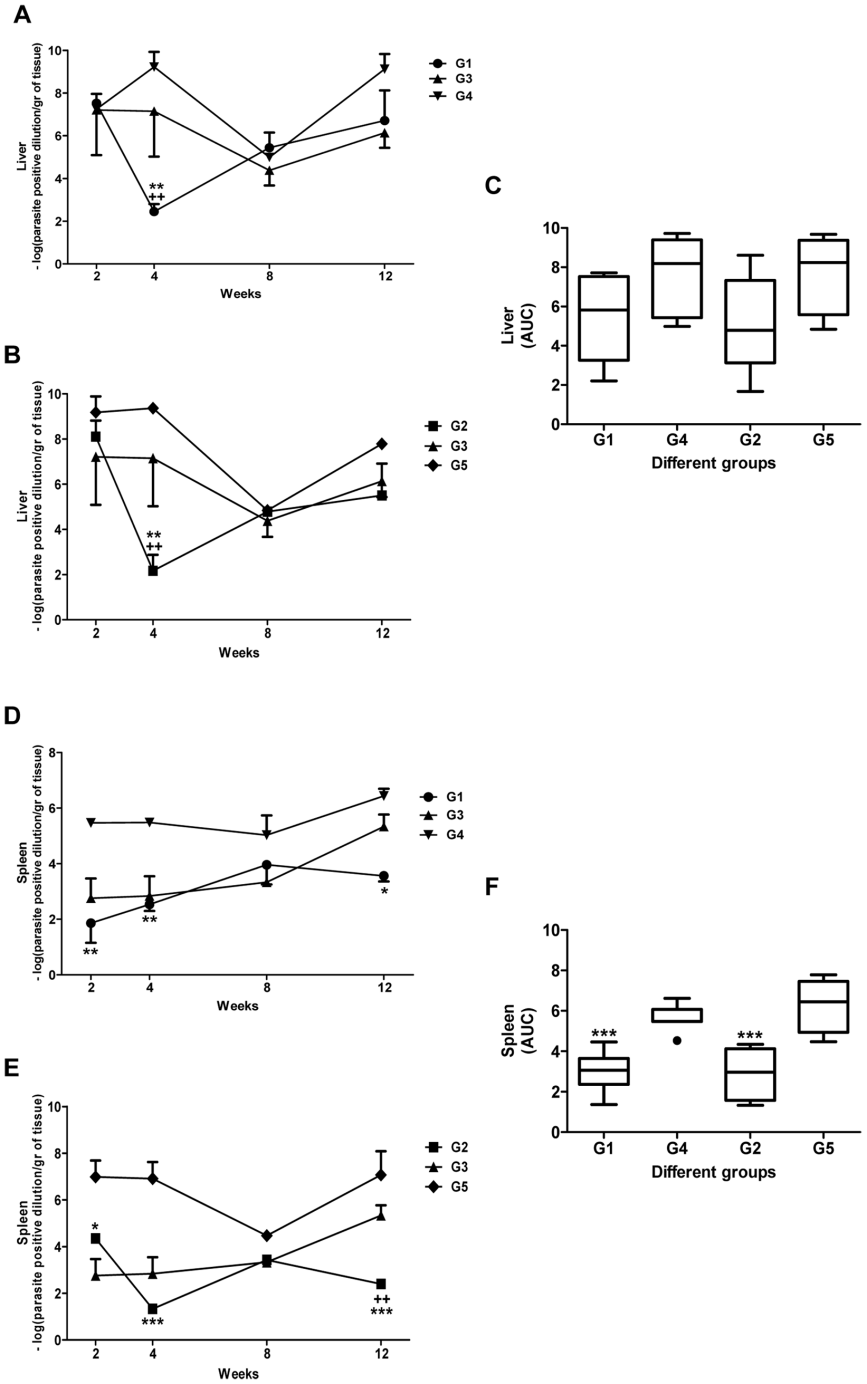
Liver and splenic parasite burden in all groups following immunization and infectious challenge with *L. infantum*. The parasite number in the liver (A, B) or spleen (D, E) was evaluated by Limiting Dilution Assay (LDA) at 2, 4, 8 and 12 weeks post-infection. The parasite burden in the liver was compared between groups G1 and G3, G4 (controls) (A) and between G2 and G3, G5 (controls) (B). Areas contained under the curves (AUC) obtained in A and B were compared between groups G1, G2 and their related controls groups G4 and G5, respectively (C). The parasite burden in spleen was compared between groups G1 and G3, G4 (controls) (D) and between G2 and G3, G5 (controls) (E). Areas contained under the curves (AUC) obtained in D and E were compared between groups G1, G2 and their related controls groups G4, G5, respectively (F). The number of two independent repeats is shown here as mean±S.E. of measures obtained from 4 mice of each group (see Materials and methods for more detail about the groups). G1 [vaccinated with DNA A2-CPA-CPB^-CTE^-cSLN (prime) and Live *L. tarentolae*-A2-CPA-CPB^-CTE^ (boost)]; G2 [vaccinated with Live *L. tarentolae*-A2-CPA-CPB^-CTE^ (prime) and Live *L. tarentolae*-A2-CPA-CPB^-CTE^ (boost)]; G3 (control PBS); G4 [(DNA vector alone (prime) and Live *L. tarentolae* wild type (boost)], and G5 [Live *L. tarentolae* wild type (prime) and Live *L. tarentolae* wild type (boost)]. Only significant differences are shown in the graphs. The asterisk sign (*) indicates the significant difference between G1 and G2 with their respective controls G4 and G5, respectively and the plus sign (+) indicates the significant difference between G1 and G2 with G3 control at the indicated time points as determined by Student's test (*p*<0.05 denoted as *, *p*<0.01 denoted as **and *p*<0.001 denoted as ***).

We first examined whether immunization with the A2-CPA-CPB^-CTE^ recombinant *L. tarentolae* was protective against *L. infantum* infectious challenge. For DNA vaccination with rA2-CPA-CPB^-CTE^, the pcDNA-A2-CPA-CPB^-CTE^ was formulated into cationic lipid particles with nanometer range (∼240–250 nm). It was shown previously that this formulation facilitates the uptake by dendritic cells and macrophages, thereby enhancing antigen expression, processing and presentation and resulting in stronger immune effects [59]. Five groups of mice were considered for immunization with two subsequent repeats as described in Materials and Methods. The results are shown as mean±S.E. of measures obtained from these two independent experiments. The degree of protection against infection was determined by weekly measurement of the parasite burden in the spleen and the liver at 2, 4, 8 and 12 weeks post-challenge and by comparing the area under the curves (AUC), as explained in the statistical analysis section in Materials and Methods. As shown in Figure 2, immunization using a DNA A2-CPA-CPB^-CTE^/Live *L. tar* A2-CPA-CPB^-CTE^ (G1) and *L. tar* A2-CPA-CPB^-CTE^/*L. tar* A2-CPA-CPB^-CTE^ (G2) prime-boosting regiments drastically (*p*<0.01) reduced the infection levels in both the liver (Fig. 2A and 2B) and the spleen (Fig. 2D and 2E) at 4 weeks after *L. infantum* challenge in contrast to the control groups (G3 to G5, n = 4 at each point/organ). The liver parasite load (Fig. 2A and 2B) of all control groups increased early following infection, reaching its maximum at 4 weeks after challenge to rapidly decline. However, the parasite burden in the vaccinated groups G1 and G2 peaked with a 4 week delay compared to the control groups. We observed a minor increase in the parasite burden in the spleen of G1 and G2 groups at week 8 but this level remained stable up to 12 weeks after infection (Fig. 2D and 2E). In the liver, there was no significant difference between all groups at 12 weeks post-infection which resulted in the no significant difference in the liver AUC of DNA/Live (G1) and Live/Live (G2) regimens with their related controls G4 and G5, respectively (Fig. 2C). Carrion *et al.* demonstrated that after intravenous injection of BALB/c mice with 10^3^, 10^5^ or 10^6^ promastigotes of *L. infantum*, VL infection would be established but the development of quantifiable immunohistological features like parasite persistence were dependent on the inoculum size [63]. According to Carrion *et al.* herein control of the hepatic infection in all groups did not result into complete clearance of the parasite in the liver (at week 12 there were still few detectable parasites present) due most likely to high inoculum (10^7^). In the spleen, the highest parasite burden in control groups (G3, G4 and G5) was observed at 12 weeks after challenge and the organ remained chronically infected (Fig. 2D and 2E). Interestingly, only the vaccinated groups G1 and especially G2, which was immunized with Live *L. tar* A2-CPA-CPB^-CTE^/Live *L. tar* A2-CPA-CPB^-CTE^ vaccine regimen, were able to control the infection as illustrated by the significant differences observed at 12 weeks after challenge in comparison to the controls (G1 with G4 and G2 with G3 and G5). Thus, the G2 vaccination regimen protects against infection better than G5 (Live *L. tar*/Live *L. tar*). Also, the spleen AUC of DNA/Live (G1) and Live/Live (G2) parasites indicate significant differences with the controls G4 and G5 (Fig. 2F). As shown by real time PCR, the parasite load in G3 is significantly higher than in the two vaccinated groups (G1 and G2) both in the liver and the spleen at 4 weeks after challenge (Fig. S2A and S2B).

### Immunization with Live Recombinant *L. tarentolae*-A2-CPA-CPB^-CTE^ Induces a Mixed Production of IFN-γ and IL-10

**Figure 3:**
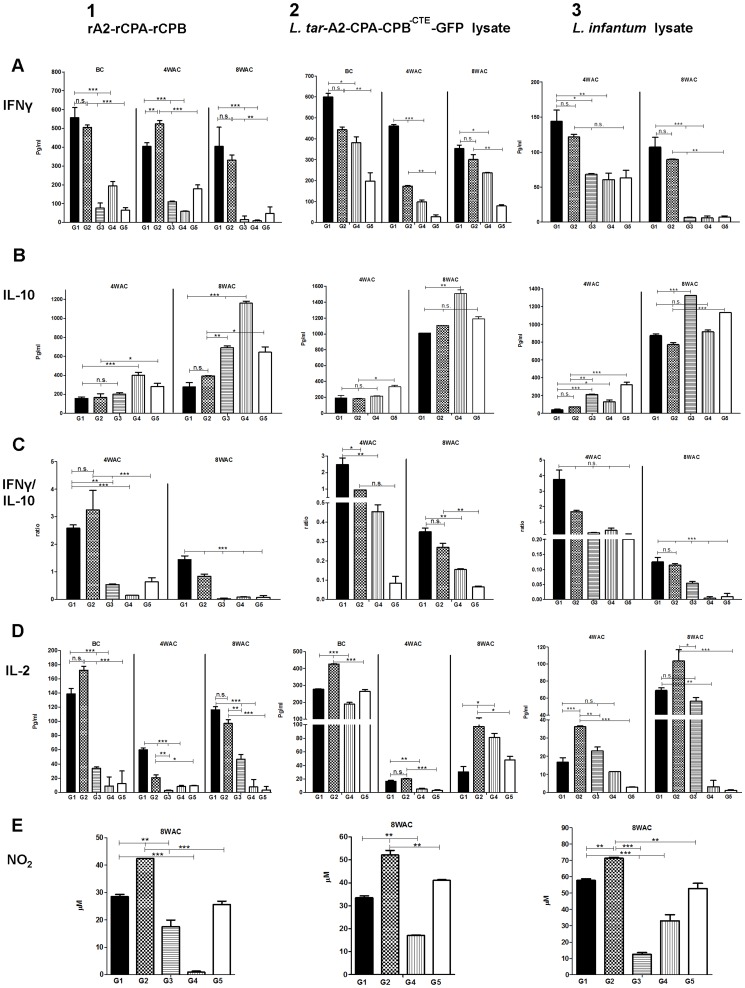
Cytokine production by splenocytes in vaccinated and control mice. IFN-γ (panel A), IL-10 (panel B), IFNγ/IL-10 ratio (panel C), IL-2 (panel D) and nitrite (NO_2_) (panel E) production after stimulation with rA2-rCPA-rCPB (column 1), F/T *L. tarentolae* A2-CPA-CPB^-CTE^-EGFP (column 2) and F/T *L. infantum* WT (column 3) as recall antigens. G1 to G5 groups are as indicated in Figure 2. Each bar represents a mean±S.E. in pg/ml (for all cytokines) or in µm for nitrite. The number of independent repeats was two and all tests were done in triplicates (number of mice per group/time point n = 3) and the results are pooled and shown as mean±S.E. of measures obtained from 6 mice in different groups. The asterisk indicates the significant difference between values at the indicated time points as determined by Student's test (*p*<0.05 denoted as *, *p*<0.01 denoted as **, *p*<0.001 denoted as *** and n.s. denoted as non significant).

The above experiments demonstrate that immunization with recombinant *L. tarentolae* expressing A2-CPA-CPB^-CTE^ confers a significant protection against *L. infantum* infection in mice. Given that production of IFN-γ is considered an important requirement for protection against *L. infantum* and that the presence of a small amount of IL-10 could promote the induction of type-1 immunity [64], we determined the levels of IFN-γ and IL-10 at different times pre- and post-challenge in cultures of splenocytes isolated from immunized and control mice in response to rA2-rCPA-rCPB, F/T *L. tar* A2-CPA-CPB^-CTE^-EGFP and F/T *L. infantum* as recall antigens (Fig. 3A–C). In addition, we analyzed the levels of IL-2 production before challenge and also at 4 and 8 weeks after challenge as well as the concentration of nitrite levels (NO_2_) only 8 weeks after challenge in the spleen of all five groups following stimulation with rA2-rCPA-rCPB, F/T *L.tar*A2-CPA-CPB^-CTE^-EGFP and F/T *L. infantum* antigens (n = 6 at each mentioned period Fig. 3D–E). As shown in Figure 3A, stimulation of splenocytes isolated from vaccinated groups G1 and G2 prior and after challenge with all three recall antigens (e.g. rA2-rCPA-rCPB, *L. tar* A2-CPA-CPB^-CTE^-EGFP lysate and *L. infantum* lysate) elicited a significantly higher IFN-γ production than control groups G3, G4 and G5. The production of IFN-γ in response to rA2-rCPA-rCPB antigens reached the highest level (524±16.4 pg/ml) at 4 weeks after challenge in G2 (Live/Live). This was significantly (*p*<0.05) different from G1 (DNA/Live) (404±19.9 pg/ml). IFN-γ levels were maintained high even 8 weeks post-challenge in both G1 (404.5±101 pg/ml) and G2 (331±27 pg/ml) (Fig. 3A). Stimulation with F/T *L. tar*A2-CPA-CPB^-CTE^-GFP recall antigen elicited also high IFN-γ production in G1 (600±22.8 pg/ml) before challenge but this was not significantly different from G2 (Fig. 3A). IFN-γ levels remained high (although slightly less than before challenge) after 4 and 8 weeks post-challenge in G1 but to a lesser extent in G2 (Fig. 3A). The production of IL-10 upon antigen stimulation at 4-week and especially at 8 weeks after challenge was lower in G1 and G2 vaccinated groups in comparison to the control groups (G3 to G5) (Fig. 3B). We further calculated the IFN-γ to IL-10 ratio for each vaccinated group as a clear indicator of successful immunization [33] (Fig. 3C). Indeed, the *Leishmania*-specific IFN-γ/IL-10 ratios upon stimulation with rA2-rCPA-rCPB and *L. tar* A2-CPA-CPB^-CTE^-GFP recall antigens were higher in G1 and G2 compared to the control G3, G4 and G5 mice both at 4 and 8 weeks after challenge. Moreover, a clear Spearman correlation was observed between the ratio of IFN-γ/IL-10 production and differences in parasite burden in liver (−.729^*^(*p* = .017)) and spleen cells (−.640^*^(*p* = .046)) stimulated with F/T *L. infantum* at 4^th^ week after challenge. This means that higher amount of IFN-γ/IL-10 ratio resulted in lower parasite burden in both spleen and liver at 4^th^ week after challenge. The IFN-γ/IL-10 ratio was drawn against the parasite burden in both spleen and liver at 4^th^ week after challenge with individual values of each group (five groups) in liver and spleen as well as their mean, and the negative slops in both graphs are in concordance with this fact (Fig. S3A and S3B).

IL-2 production that is important for lymphocyte proliferation was higher in G1 and G2 than in control groups (Fig. 3D). Significant differences were seen in the amount of IL-2 production for G2 at all time points in response to different recall antigens. Similar levels of cytokines were produced following exposure to ConA in all groups (data not shown).

The highest amount of nitric oxide, which is essential for killing parasites inside infected macrophages, was observed with the Live/Live modality vaccinated mice (G2) upon stimulation with rA2-rCPA-rCPB, F/T *L. tar*A2-CPA-CPB^-CTE^-GFP and F/T *L. infantum* antigens at 8 weeks after challenge (Fig. 3E).

### Immunization with Live Recombinant *L. tarentolae*-A2-CPA-CPB^-CTE^ Induces both IgG1 and IgG2a Responses

**Figure 4:**
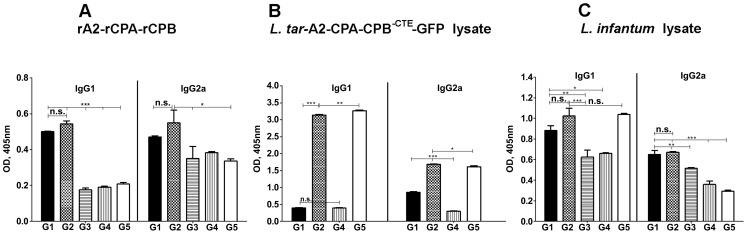
Analysis of the specific humoral response induced in vaccinated and control mice before and after challenge. Mice were bled after two vaccinations before challenge and 5 weeks after challenge. Sera were obtained from individual mice from each group and pooled (n = 15). G1 to G5 groups are as indicated in Figure 2. Before challenge sera were tested for anti rA2-rCPA-rCPB (A) and F/T *L. tarentolae* A2-CPA-CPB^-CTE^-EGFP (B) and after challenge sera were tested for anti F/T *L. infantum* (C) antibodies by an isotype-specific ELISA. The asterisk indicates the significant difference between values at the indicated time points as determined by Student's test (*p*<0.05 denoted as *, *p*<0.01 denoted as **, *p*<0.001 denoted as *** and n.s. denoted as non significant).

**Figure 5:**
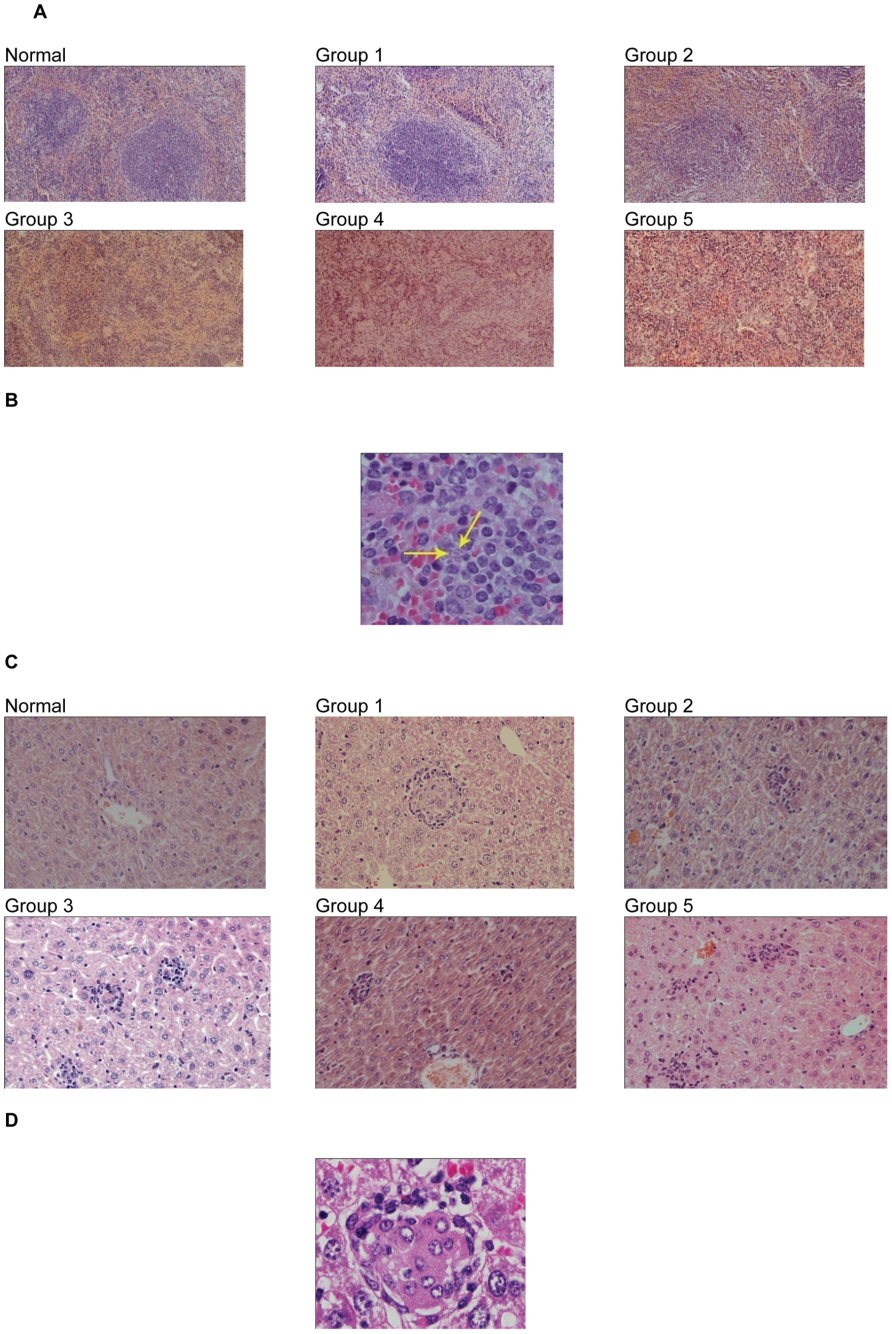
Liver and spleen histological sections stained with hematoxylin and eosin (H&E). (A) Splenic architecture of normal (non-immunized) mice in comparison with groups G1 to G5 as indicated in Figure 2 at 4^th^ week post-infection with 10^7^ *L. infantum* stationary promastigotes. Disruption of the splenic architecture accompanied by lymphoid depletion could be seen in control mice infected with 10^7^ *L. infantum* promastigotes after the first month. (B) Presence of parasites in the spleen tissue of non-vaccinated groups at 4^th^ week after challenge with 10^7^ *L. infantum*. (C) The liver granuloma reaction at 8 weeks following *L. infantum* challenge infection in all groups compared to the non-infected mice. (D) Mature granuloma assembly in control group 3, resulting in the attraction of lymphocytes and monocytes at 14^th^ week after challenge The number of independent repeats was two and number of mice per group/time point n = 2.

To compare IgG isotypes in different groups, all sera were assayed by ELISA before (Fig. 4A–B) and 5 weeks after (Fig. 4C) challenge. As shown in Figures 4A and 4C, both rA2-rCPA-rCPB and F/T *L. infantum* specific IgG1 and IgG2a were higher in vaccinated groups G1 and G2 compared to the control groups. Although G1 shows higher amount of rA2-rCPA-rCPB specific IgG2a, there were no significant differences with control groups. Also, increased amount of F/T *L. infantum* specific IgG1 was seen in G5 at 5 weeks after challenge. Interestingly, in G2 vaccinated mice with Live *L.tar* A2-CPA-CPB^-CTE^-GFP/Live *L.tar* A2-CPA-CPB^-CTE^-GFP, a higher amount of F/T *L. tar* A2-CPA-CPB^-CTE^-GFP specific IgG1 and IgG2a was detected in comparison to G1 and G4 vaccinated with DNA/Live modality (Fig. 4B).

### Histopathological Studies in the Vaccinated Mice

Two animals from all groups at each round of experiment were sacrificed at specific time points (4, 8, and 14 weeks) after challenge to collect liver, spleen and bone marrow samples for histopathological analysis. In spleen, splenic cords were normal and somewhat thickened in vaccinated groups but were thin and in some cases were disappeared in other groups. Normal follicle formation of the spleen was noted in G1 and G2 in 4^th^ and 8^th^ week but the normal follicles were disappeared in the other groups (G3, G4 and G5) (Fig. 5A). Absence of follicles and derangement of splenic cords disfigured the total architecture of the spleen. In total of 18 non-vaccinated mice (G3, G4 and G5) during 4, 8 and 14 weeks after challenge, the architecture was completely distorted in 12, minimally changed in 3 and unchanged in 3 mice. However, in vaccinated groups (G1 and G2, total of 12 mice), the architecture was relatively unchanged in 9 and minimally changed in 3 mice (Fig. 5A). Spleen granuloma formation was noted only in 3 mice of non-vaccinated cases in 4^th^ week and not seen later. We could not find parasites in the spleen in vaccinated groups (G1 and G2) but they could be seen in nearly all non-vaccinated groups (G3, G4 and G5, Fig. 5B).

In the liver, mononuclear cell infiltration in portals was more prominent in non-vaccinated groups at week 14^th^. Plasma cells were only seen in these groups. Interface hepatitis was not a significant finding. Parasites in the portals were only seen in non-vaccinated groups (G3 and G5) in 8^th^ week (data not shown).

**Figure 6:**
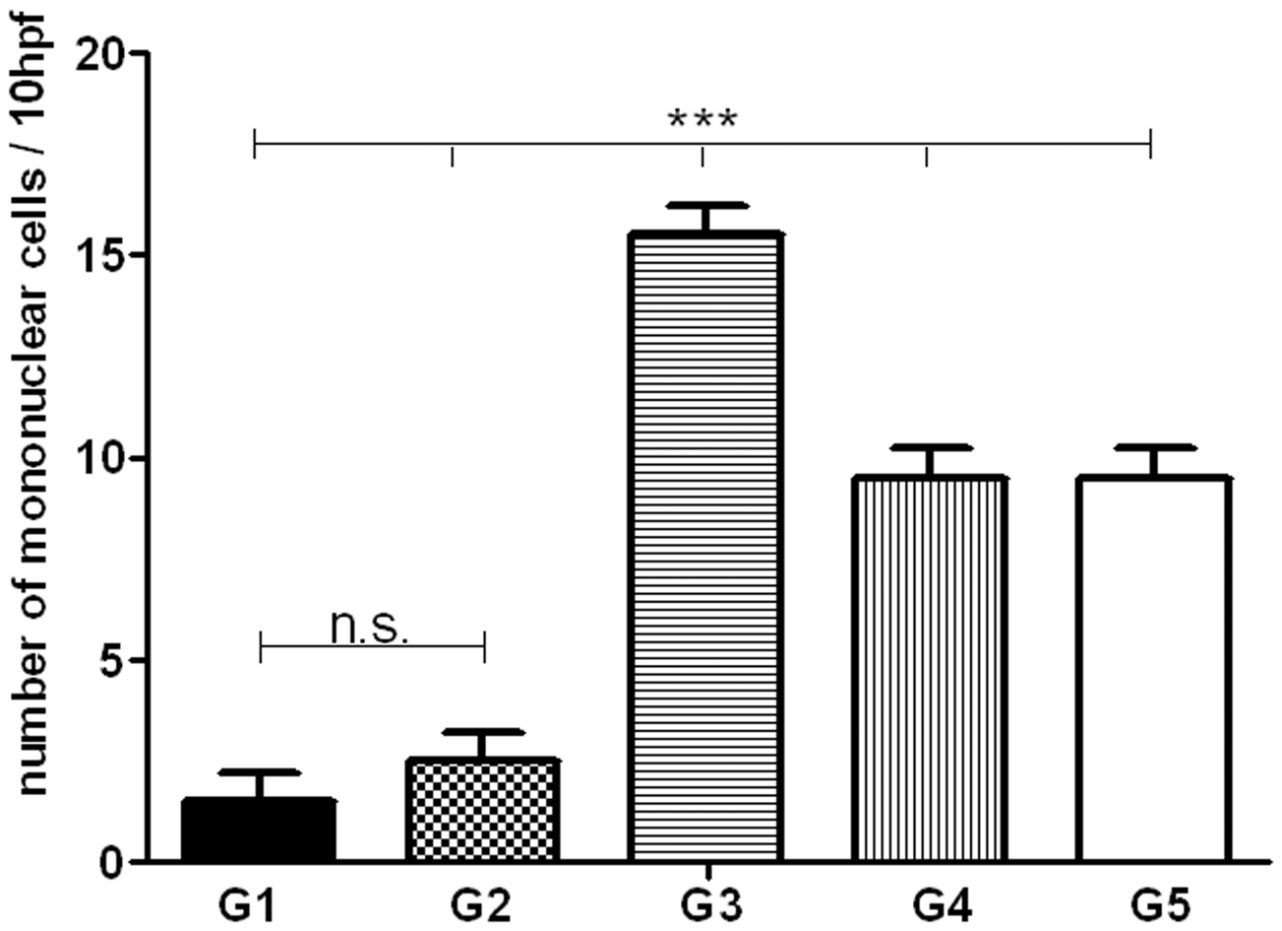
Degree of inflammatory cell infiltration in liver parenchyma of all groups at 4 weeks after challenge. G1 to G5 groups are as indicated in Figure 2. The number of independent repeats was two and all tests were done in duplicate (number of mice per group/time point n = 2) and the results are pooled and shown as mean±S.E. of measures obtained from 4 mice of each group. The asterisk indicates the significant difference between values at the indicated time points as determined by Student's test (*p*<0.05 denoted as *, *p*<0.01 denoted as **, *p*<0.001 denoted as *** and n.s. denoted as non significant).

In all groups, lobular inflammation was seen at 4^th^ week, increased significantly in 8^th^ week and decreased at week 14. The severity of this inflammatory response at 4^th^ week was higher in G3 compared to the other groups (15–16/10 hpf (high power field) v.s 2–4/10 hpf of vaccinated groups, p<0.05) (Fig. 6). Inflammatory cell infiltration was seen in the liver parenchyma in groups 1, 2, 3, 4 and 5 at 4^th^ week after challenge (Fig. S4). Portal granuloma formation (either mature or immature) was seen as early as 8^th^ week in non vaccinated group, but in parenchyma of all groups they were seen at this time and persisted until 14^th^ week (Fig. 5C and 5D). Parasite in the parenchyma was visualized only in one out of 12 mice of vaccinated groups but it was seen in 11 out of 18 non-vaccinated mice. Occurrence of cholestasis was noted at 8^th^ week with no predilection to any group (data not shown). There was no significant difference in neutrophil infiltration, fatty changes, giant cell formation, and kupffer cell hyperplasia between groups (data not shown). Liver cell necrosis was minimal and no significant regenerative changes were noted (data not shown). No parasites were found in bone marrow macrophages and no significant changes in bone marrow of all examined mice were observed (data not shown).

## Discussion

Although preventive vaccines are recognized as the best and most cost-effective protection measure against pathogens, no effective vaccines are available to control leishmaniasis. *Leishmania* vaccine development has been proven to be a difficult and challenging task and is hampered by an inadequate knowledge of disease pathogenesis, the complexity of immune responses needed for protection, and the cost of vaccine development [16], [65]. Immunization against experimental visceral leishmaniasis in murine models has been reported to be more difficult to achieve than for the cutaneous form. This may be due to the more complex situation in immunopathology of murine infection with VL species. In particular, the outcome of VL infection in mice does not depend on the Th1 versus Th2 subset expansion [66], [67]. Opposite to *L. major* murine model, this dichotomy seems inadequate in human and murine forms of VL. Type 1 immune responses are suppressed by IL-10 and TGF-β [68]. Based on recent data about T reg expansion during infection, Foxp3 expressing CD4^+^CD25^+^ T cells were found responsible for TGF-β and CD4^+^CD25^−^Foxp3^−^ T cells for IL-10 production [69]. In some areas, the inoculation of infectious material isolated from cutaneous lesions or cultured virulent promastigotes (a process called leishmanization) in hidden parts of uninfected individuals has been used to prevent further lesions [70]. These individuals develop a strong immunity to reinfection, sometimes only after unpleasant clinical episodes. Thus, there is a general consensus among *Leishmania* vaccine researchers that parasite persistence and exposure to complex antigens in the right context over time may be important for effective protective response and could be achieved by live attenuated parasites immunization. However, reversion of these parasites to the virulent form restricts their use [71]. Early attempts for the development of attenuated strains (e.g. obtained through long-term *in vitro* culture, γ-irradiation, selection for temperature sensitivity, or chemical mutagenesis) did not elicit a protective immunity [72]. On the other hand, manipulation of the *Leishmania* genome through discovery of new genes involved in parasite growth and survival revives the potential of a live attenuated parasite vaccine with reduced risk of reversion. In this context, several successful attenuated lines of *L. donovani*, such as a partial knockout of *A2-A2rel* gene clusters with attenuated virulence in mice [73], a biopterin transporter knockout with reduced infectivity and induced protection against challenge [35] and a centrin (LdCen1^−***/***−^) knockout with reduced parasite survival in macrophages [74] have been generated. Using these live attenuated *Leishmania* vaccines, which most closely mimic the natural course of infection, could be very beneficial. On the other hand, return back to virulence may occur, and there is an increasing need to develop new safer live vaccine vectors that are capable of enhancing antigen presentation and eliciting potent immune responses without the risk of disease development in humans. Consequently, a live nonpathogenic to humans parasitic vector consisting of a lizard parasitic protozoan, *L. tarentolae*, has been introduced by Breton *et al.* as a candidate vaccine against visceral leishmaniasis [38]. We have demonstrated previously that a recombinant *L. tarentolae* strain expressing the *L. donovani A2* gene elicited a strong protective immunity against virulent *L. infantum* challenge [42]. We have also showed that vaccination with *L. infantum* cysteine proteinases type I and II as part of a prime-boost strategy induced a strong parasite-specific Th1 response and conferred protection against parasite challenge [51].

As a follow-up of our previous work, the objective of this study was to generate a recombinant *L. tarentolae* strain stably expressing three candidate antigens as part of a tri-gene fusion, including the *L. donovani A2* and *L. infantum CPA* and *CPB* genes and to evaluate its potential as a candidate live vaccine against VL using different prime-boost vaccine modalities. Immunization of BALB/c mice with recombinant *L. tarentolae* A2-CPA-CPB^-CTE^ as a DNA/Live or a Live/Live prime-boost regimen (groups G1 and G2) elicited a significant protective immunity against a high dose virulent *L. infantum* challenge. Moreover, mice immunized with these modalities did control the parasite propagation both in the liver and the spleen at 4 weeks but also 12 weeks after challenge. It is worth mentioning that tissue parasite loads were measured with the limiting dilution assay that is semi-quantitative and may give inconsistent results due to medium and incubation conditions. Hence, for more accurate measurements of parasite burden considering both LDA and qPCR is highly recommended. Therefore at week 4 after infectious challenge, we also confirmed the quantity differences between vaccinated groups (G1 and G2) and PBS control group (G3) by qPCR in both liver and spleen. Interestingly, the obtained results for this specific time point are in accordance with semi-quantitative LDA.

In our experimental system, immunization of BALB/c mice with recombinant *L. tarentolae* A2-CPA-CPB^-CTE^ revealed the induction and secretion of IFN-γ and IL-10, although IFN-γ was much higher than IL-10. Determining the main source of IL-10 production has some beneficial results for understanding mechanisms of resistance and susceptibility to VL. Here, in immunized G1 and G2 groups, we detected high IL-10 production, especially at 8 weeks after challenge, upon stimulation with *L. infantum* lysate as a recall antigen. The sources of IL-10 in human VL have not been defined yet. IL-10 can be produced by several cell types such as monocytes, macrophages, B cells and CD4^+^ T cells. It has been described that several subpopulations of IL-10 producing CD4^+^ T cells have the ability to inhibit the response of other T cells. One of the important sources of IL-10 in the suppression of anti-leishmanial immunity in human VL is T regulatory (T reg) cells that arise from CD25^−^Foxp3^−^ T cells [75]. Also, there are some CD3^+^ CD25^−^ depleted cells that produce both IFN-γ and IL-10 cytokines. Simultaneous production of IFN-γ and IL-10 by human T cell clones can be induced by IL-12 [76]. In addition, IL-10 produced by adaptive T reg cells may be preferentially required to control tissue damage in sites of strong inflammation associated with antimicrobial responses [77].

The IFN-γ/IL-10 ratio in splenocytes stimulated with rA2-rCPA-rCPB from mice immunized with the recombinant *L. tarentolae* A2-CPA-CPB^-CTE^ was ∼5.1- to 11.2-fold higher at 4 and 8 weeks post-challenge than mice immunized with wild type *L. tarentolae* only. The same result was obtained with splenocytes stimulated with other F/T antigens. Also, histopathological studies confirmed the Spearman correlation between the ratio of IFN-γ/IL-10 production and differences in parasite burden in liver and spleen cells as no parasites were seen in the liver parenchyma and spleen at 4^th^ week post-challenge in the vaccinated groups G1 and G2, whereas they were easily detected in the non-vaccinated control groups. In addition, the level of both IgG1 and IgG2a at different time points before and after challenge was not significantly different between the vaccinated groups. Thus, cytokine data along with antibody titration indicated that vaccination with DNA/Live *L. tarentolae* A2-CPA-CPB^-CTE^ and Live/Live *L. tarentolae* A2-CPA-CPB^-CTE^ induce a clearly stronger Th1 response.

Although it has been suggested that the control of splenic parasite burden is likely to be mediated through a NO-dependent or a NO-independent pathway [78], in this report we showed that *L. tarentolae* A2-CPA-CPB^-CTE^, especially when administered as a Live/Live modality, induces a strong humoral and cellular immune response in addition to NO generation in response to rA2-rCPA-rCPB, F/T *L. tarentolae* A2-CPA-CPB^-CTE^ and F/T *L. infantum* specific stimulators at 8 weeks after infectious challenge with *L. infantum*. Possibly, the cumulative effect of IFN-γ along with iNOs-mediated parasite killing is responsible for this response. Furthermore, it has been reported that one of the major factors contributing to healing of leishmaniasis is the development of strong CMI (cell-mediated immunity) response like IFN-γ and NO production [66], [68], [79]. Therefore, higher IFN-γ and NO production in vaccinated groups G1 and G2 suggests a fine correlation between CMI and resistance to VL infection. The generation of NO in stimulated cells with rA2-rCPA-rCPB, F/T *L. tarentolae* A2-CPA-CPB^-CTE^ and F/T *L. infantum* also supports the up-regulation of inducible NO synthase by TH1 cells and confirms that the NO-mediated macrophage effector mechanism is critical in the control of parasite replication in these animals. Our results showed that the nitrite (NO_2_) levels are in direct correlation with IFN-γ production in these groups and that higher production of IFN-γ was obtained after 56 days of infection, indicating a possible control of the parasites' persistence and suggesting that the control of *L. infantum* infection may occur by a NO-dependent pathway.

It has been described previously that during the early stages of visceral infection in BALB/c mice, parasites multiply in large numbers in the liver; however, once the infection becomes chronic, hepatic parasite loads tend to decrease, while parasitism in the spleen and BM tends to increase [63]. Pathology is mediated by the direct loss of specific cell populations and changes to the local tissue microenvironments that ultimately decrease the induction of effective immune responses and by the inability of splenic macrophage populations to generate leishmanicidal mechanisms or to recruit appropriate cells for eliminating the parasite. Furthermore, the intensity of pathological changes in the visceral organs of BALB/c mice can vary depending on the initial inoculum size [63], [80]. Pathological studies showed that after infection with 10^7^ *L. infantum* promastigotes, control groups exhibited severe histopathological alterations in both the spleen and liver at the peak of parasite burden. Once in the liver, the development of cell-mediated immune responses is essential for the clearance of *L. infantum* parasites. In contrast, the spleen ultimately becomes the site of parasite persistence [63], [81], [82], suggesting that it is more susceptible to *L. infantum* infection than the liver [83]. Interestingly, the leishmanicidal efficacy of hepatic granulomas is dependent on their degree of maturation [63], [84], [85]. Among these alterations, we detected the appearance of granulomas in different maturation stages and giant cell granulomas in amastigotes in the liver of all groups infected with *L. infantum*, which results in liver parasite clearance. However, disruption of the splenic architecture accompanied by lymphoid depletion only in control groups results in spleen parasite persistence.

Overall, our results on cytokine production, humoral responses, parasite burden and histopathological studies support that immunization with the novel recombinant *L. tarentolae* A2-CPA-CPB^-CTE^ candidate vaccine protects mice against visceral leishmaniasis when administered as a prime-boost modality more than *L. tarentolae* [38] or *L. tarentolae* A2 [42]. The next step will be to determine the long-term memory protection in mice or hamsters and to evaluate the effectiveness of this promising live vaccine against *L. infantum* in dogs as an important outbreed animal model for VL research.

## Supporting Information

Figure S1 **Generation of a recombinant** ***L. tarentolae*** **strain expressing the A2-CPA-CPB^-CTE^-EGFP tri-fusion gene.** (A) Schematic representation of the linearized pLEXSY-A2-CPA-CPB^-CTE^-EGFP construct containing two regions of homology to the rRNA locus of *L. tarentolae* 5′*ssu* and 3′*ssu* for genomic integration by homologous recombination following transfection into *L. tarentolae*. (B) PCR analysis to confirm integration of the A2-CPA-CPB^-CTE^-EGFP cassette into the *ssu* locus (lane 1). Wild type *L. tarentolae* was used as a negative control (lane 2). (C) Southern blot hybridization of DNA extracted from *L. tarentolae* promastigotes and digested with *Bgl*II/*Not*I with a GFP-labeled probe. Lanes 1, *L. tarentolae* wild type; 2, a 2.8-kb region of A2-CPA-CPB^-CTE^-EGFP; and 3, a<1-kb region of EGFP for *L. tarentolae*-EGFP as a positive control. (D) RT-PCR analysis of *A2*, *CPA*, *CPB^-CTE^* and *EGFP* genes from cDNA of *L. tarentolae*-A2-CPA-CPB^-CTE^-EGFP. The 708 bp band represents the *A2* PCR product on cDNA template (lane 1), a 687 bp band represents the *CPA* PCR product (lane 3), a 639 bp band represents the *CPB* ^-CTE^ PCR product (lane 5), and a 735 bp band represents the *EGFP* PCR product (lane 7). No bands were detected for the PCR reaction on RNA templates for *A2*, *CPA*, *CPB^-CTE^* and *EGFP* genes, respectively in lanes 2, 4, 6 and 8.
(TIF)

Figure S2 **Quantification of parasites in liver and spleen at 4 weeks after challenge by real time PCR assays.** The parasite number in the liver (A) or spleen (B) was evaluated by Real time PCR at 4 weeks post- infection for vaccinated groups (G1 and G2) and PBS control group (G3). G1 [vaccinated with DNA A2-CPA-CPB^-CTE^-cSLN (prime) and Live *L. tarentolae*-A2-CPA-CPB^-CTE^ (boost)]; G2 [vaccinated with Live *L. tarentolae*-A2-CPA-CPB^-CTE^ (prime) and Live *L. tarentolae*-A2-CPA-CPB^-CTE^ (boost)] and G3 (control PBS) groups. The number of independent repeats was two and all tests were done in duplicate (number of mice per group/time point n = 2). The result is shown here as mean±S.E. of measures obtained from 4 mice of each group. The asterisk indicates the significant difference between values at the indicated time points as determined by Student's test (*p*<0.05 denoted as *, *p*<0.01 denoted as **, *p*<0.001 denoted as *** and n.s. denoted as non significant).
(TIF)

Figure S3 **IFN-γ/IL-10 ratio against parasite burden in liver and spleen at 4^th^ week after challenge.** IFN-γ/IL-10 ratio inversely correlates with the parasite burden at 4 weeks after challenge with individual values of each group (five groups) in liver (A) and spleen (B) (each data series represented by different colors). Inset graphs in both A and B shows their mean with significant *p* value (*p*<0.05) and r^2^. Groups G1 [vaccinated with DNA A2-CPA-CPB^-CTE^-cSLN (prime) and Live *L. tarentolae*-A2-CPA-CPB^-CTE^ (boost)]; G2 [vaccinated with Live *L. tarentolae*-A2-CPA-CPB^-CTE^ (prime) and Live *L. tarentolae*-A2-CPA-CPB^-CTE^ (boost)]; G3 (control PBS); G4 [DNA vector alone (prime) and Live *L. tarentolae* wild type (boost)], and G5 [Live *L. tarentolae* wild type (prime) and Live *L. tarentolae* wild type (boost)].
(TIF)

Figure S4 **Inflammatory cell infiltration in the liver parenchyma of all groups at 4^th^ week after challenge.** The liver parenchyma were stained with hematoxylin and eosin (H&E). G1 to G5 groups are as indicated in Figure S3. No evidence of mononuclear cell infiltration is observed in parenchyma of G1. Lobular inflammation is very mild and is seen in the parenchyma of G2, but severe lobular infiltration of mononuclear cells and many foci of inflammation are seen in the parenchyma of groups 3, 4 and 5. The result of two independent repeats was pooled and number of mice in total per group is n = 4.
(TIF)
