# Supplementary figures and images for: Development of Novel Prime-Boost Strategies Based on a Tri-Gene Fusion Recombinant L. tarentolae Vaccine against Experimental Murine Visceral Leishmaniasis
Source: PLoS Negl Trop Dis. 2013 Apr 18;7(4):e2174. doi: 10.1371/journal.pntd.0002174 (PMC3630202; doi:10.1371/journal.pntd.0002174)

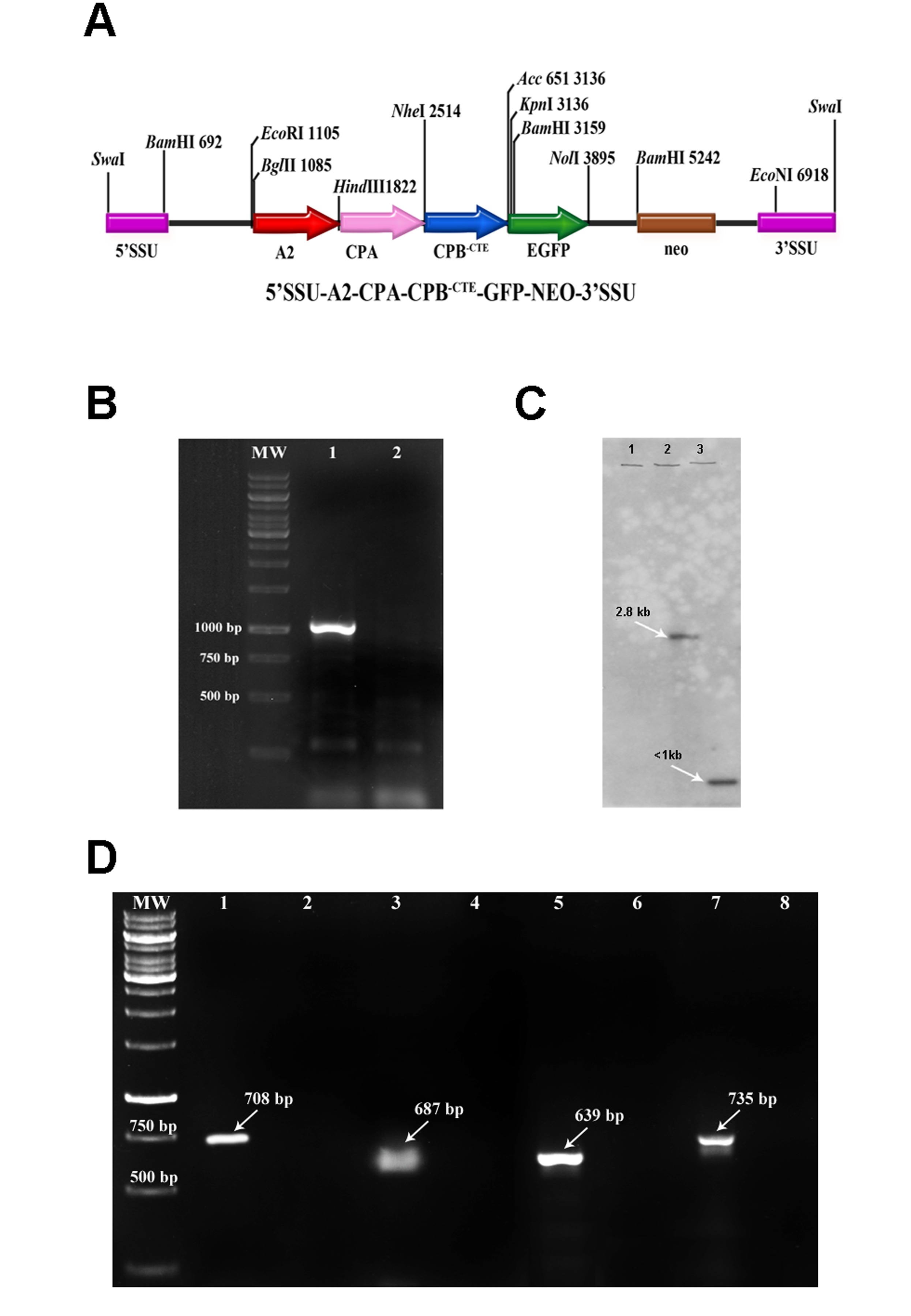

Supplement: Figure S1 — Generation of a recombinant L. tarentolae strain expressing the A2-CPA-CPB-CTE-EGFP tri-fusion gene. (A) Schematic representation of the linearized pLEXSY-A2-CPA-CPB-CTE-EGFP construct containing two regions of homology to the rRNA locus of L. tarentolae 5′ssu and 3′ssu for genomic integration by homologous recombination following transfection into L. tarentolae. (B) PCR analysis to confirm integration of the A2-CPA-CPB-CTE-EGFP cassette into the ssu locus (lane 1). Wild type L. tarentolae was used as a negative control (lane 2). (C) Southern blot hybridization of DNA extracted from L. tarentolae promastigotes and digested with BglII/NotI with a GFP-labeled probe. Lanes 1, L. tarentolae wild type; 2, a 2.8-kb region of A2-CPA-CPB-CTE-EGFP; and 3, a<1-kb region of EGFP for L. tarentolae-EGFP as a positive control. (D) RT-PCR analysis of A2, CPA, CPB-CTE and EGFP genes from cDNA of L. tarentolae-A2-CPA-CPB-CTE-EGFP. The 708 bp band represents the A2 PCR product on cDNA template (lane 1), a 687 bp band represents the CPA PCR product (lane 3), a 639 bp band represents the CPB -CTE PCR product (lane 5), and a 735 bp band represents the EGFP PCR product (lane 7). No bands were detected for the PCR reaction on RNA templates for A2, CPA, CPB-CTE and EGFP genes, respectively in lanes 2, 4, 6 and 8. (TIF) [file pntd.0002174.s001.tif]

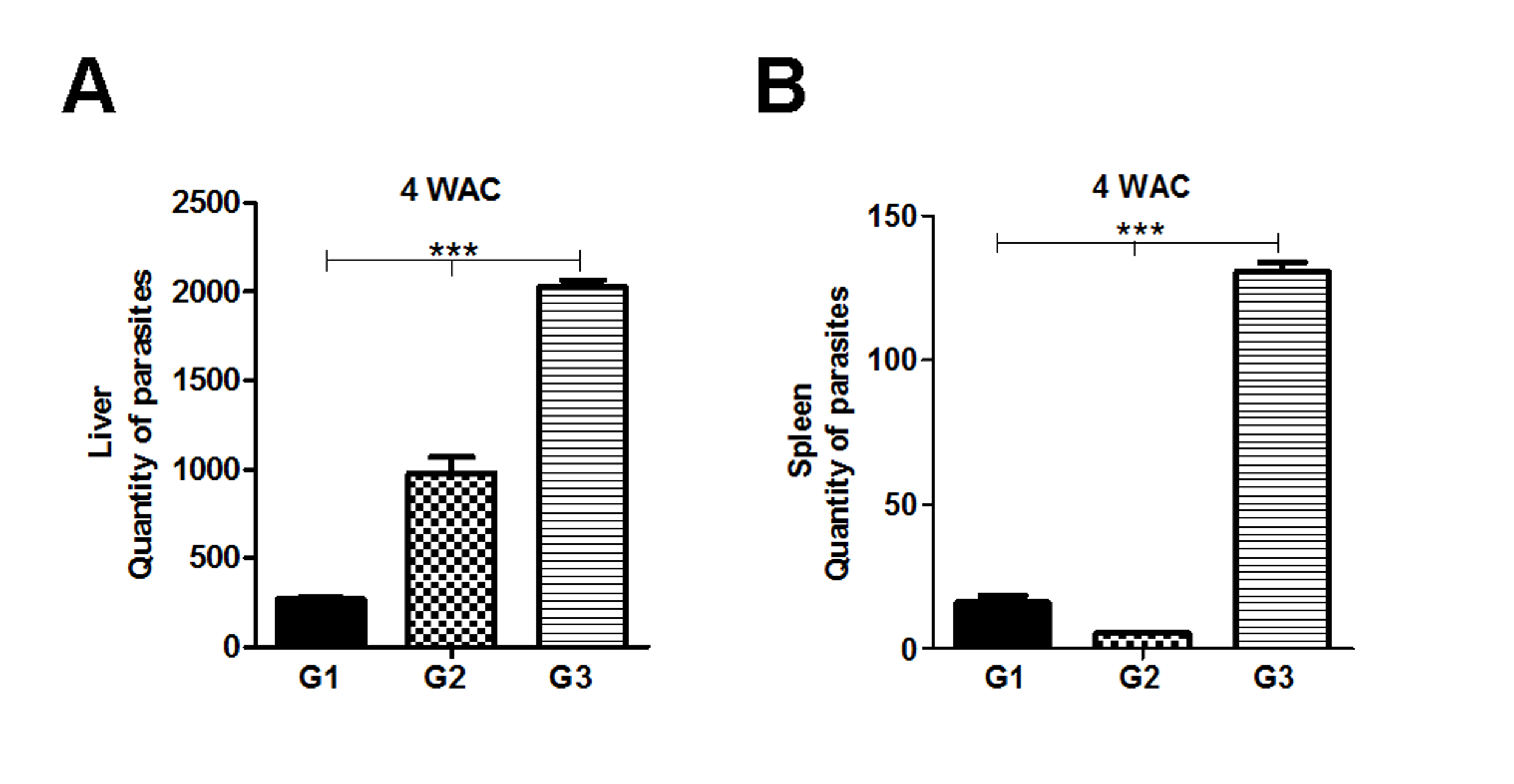

Supplement: Figure S2 — Quantification of parasites in liver and spleen at 4 weeks after challenge by real time PCR assays. The parasite number in the liver (A) or spleen (B) was evaluated by Real time PCR at 4 weeks post- infection for vaccinated groups (G1 and G2) and PBS control group (G3). G1 [vaccinated with DNA A2-CPA-CPB-CTE-cSLN (prime) and Live L. tarentolae-A2-CPA-CPB-CTE (boost)]; G2 [vaccinated with Live L. tarentolae-A2-CPA-CPB-CTE (prime) and Live L. tarentolae-A2-CPA-CPB-CTE (boost)] and G3 (control PBS) groups. The number of independent repeats was two and all tests were done in duplicate (number of mice per group/time point n = 2). The result is shown here as mean±S.E. of measures obtained from 4 mice of each group. The asterisk indicates the significant difference between values at the indicated time points as determined by Student's test (p<0.05 denoted as *, p<0.01 denoted as **, p<0.001 denoted as *** and n.s. denoted as non significant). (TIF) [file pntd.0002174.s002.tif]

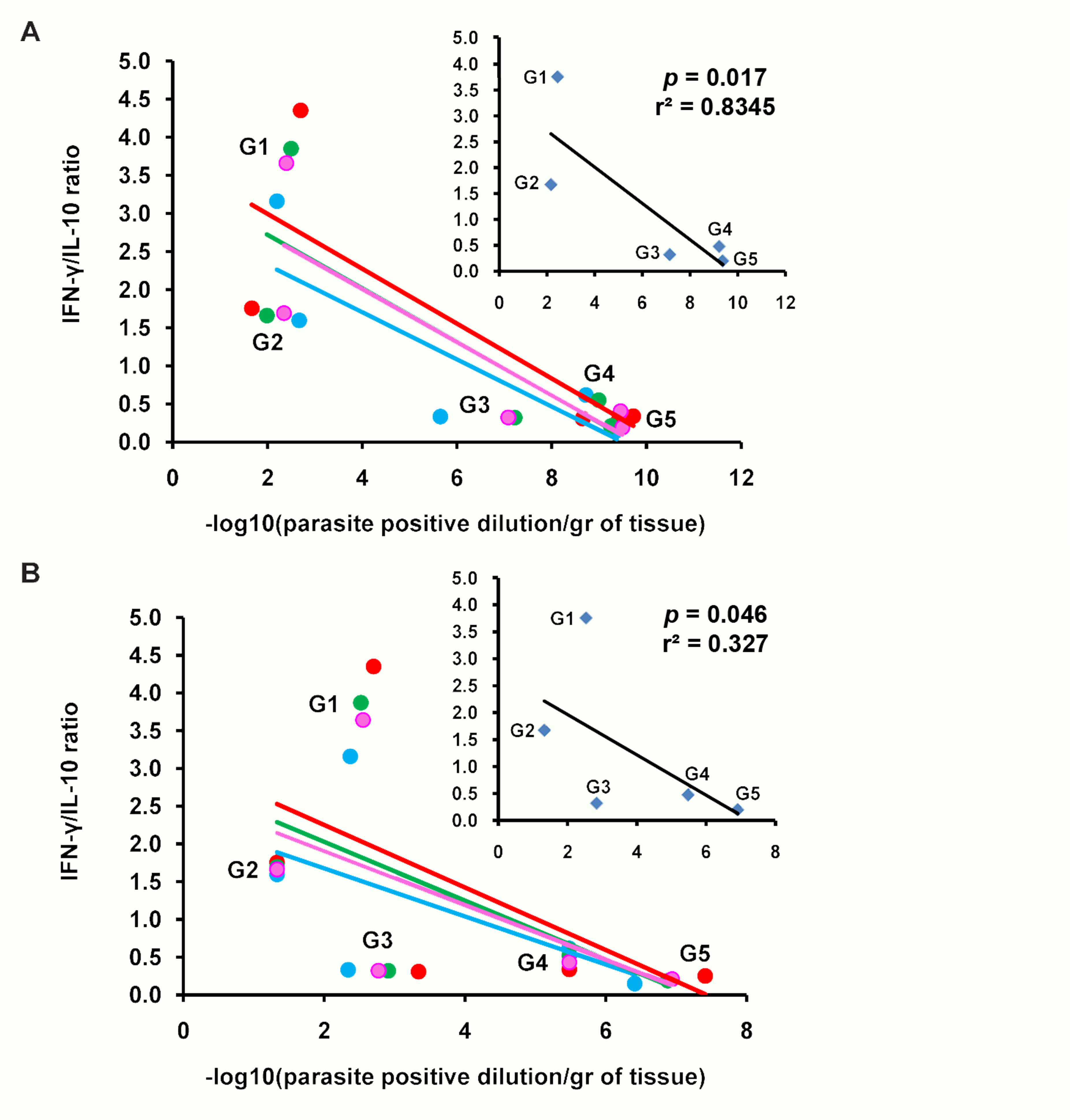

Supplement: Figure S3 — IFN-γ/IL-10 ratio against parasite burden in liver and spleen at 4th week after challenge. IFN-γ/IL-10 ratio inversely correlates with the parasite burden at 4 weeks after challenge with individual values of each group (five groups) in liver (A) and spleen (B) (each data series represented by different colors). Inset graphs in both A and B shows their mean with significant p value (p<0.05) and r2. Groups G1 [vaccinated with DNA A2-CPA-CPB-CTE-cSLN (prime) and Live L. tarentolae-A2-CPA-CPB-CTE (boost)]; G2 [vaccinated with Live L. tarentolae-A2-CPA-CPB-CTE (prime) and Live L. tarentolae-A2-CPA-CPB-CTE (boost)]; G3 (control PBS); G4 [DNA vector alone (prime) and Live L. tarentolae wild type (boost)], and G5 [Live L. tarentolae wild type (prime) and Live L. tarentolae wild type (boost)]. (TIF) [file pntd.0002174.s003.tif]

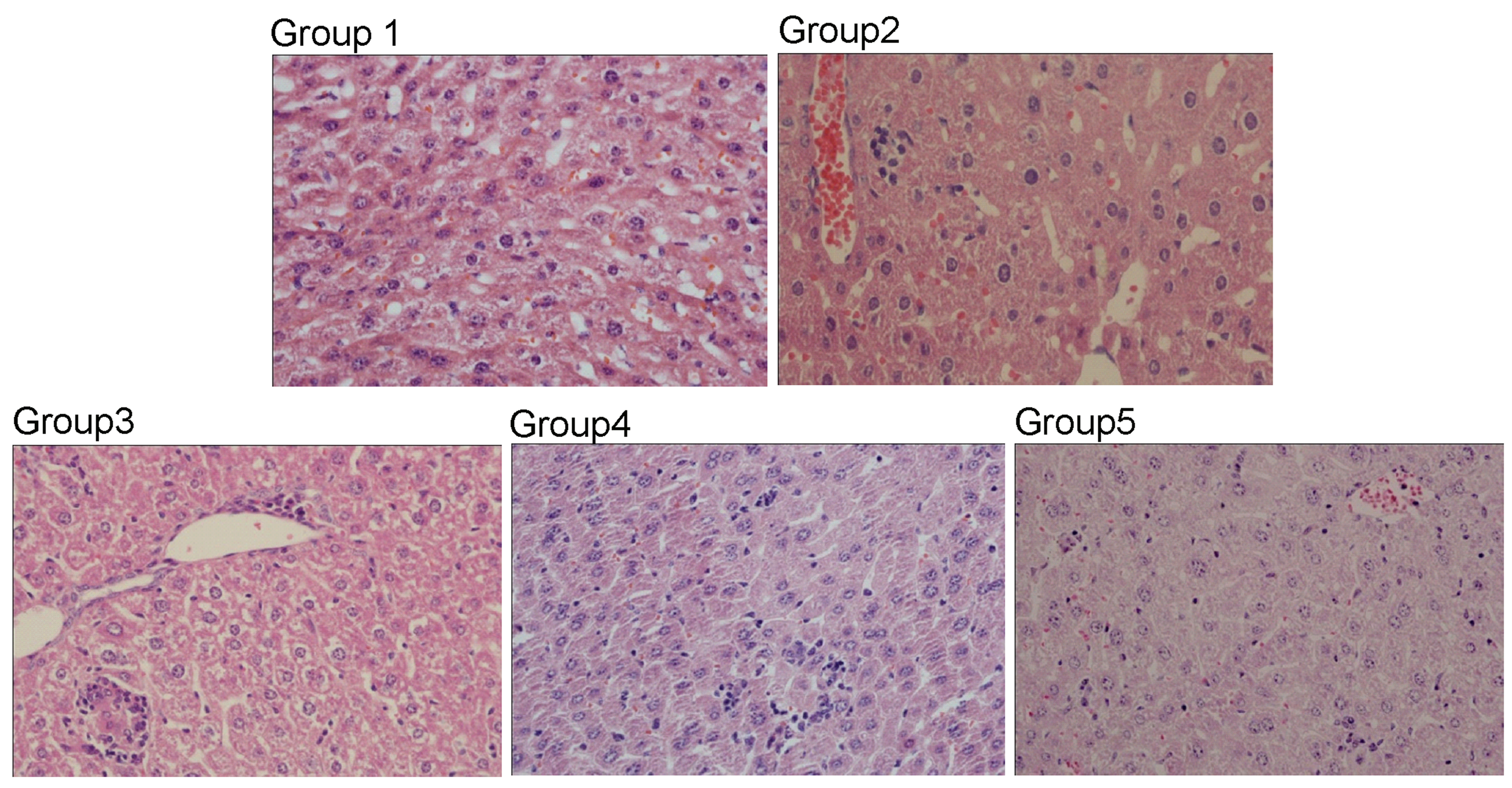

Supplement: Figure S4 — Inflammatory cell infiltration in the liver parenchyma of all groups at 4th week after challenge. The liver parenchyma were stained with hematoxylin and eosin (H&E). G1 to G5 groups are as indicated in Figure S3. No evidence of mononuclear cell infiltration is observed in parenchyma of G1. Lobular inflammation is very mild and is seen in the parenchyma of G2, but severe lobular infiltration of mononuclear cells and many foci of inflammation are seen in the parenchyma of groups 3, 4 and 5. The result of two independent repeats was pooled and number of mice in total per group is n = 4. (TIF) [file pntd.0002174.s004.tif]
